# Proteomic analysis of the secretome of *Leishmania donovani*

**DOI:** 10.1186/gb-2008-9-2-r35

**Published:** 2008-02-18

**Authors:** J Maxwell Silverman, Simon K Chan, Dale P Robinson, Dennis M Dwyer, Devki Nandan, Leonard J Foster, Neil E Reiner

**Affiliations:** 1 Department of Medicine (Division of Infectious Diseases), University of British Columbia, Faculty of Medicine, 2733 Heather St, Vancouver, British Columbia, V5Z 3J5, Canada; 2 Vancouver Coastal Health Research Institute, 2647 Willow St. Vancouver, British Columbia, V5Z 3P1, Canada; 3 Department Microbiology and Immunology, University of British Columbia, Faculty of Science, 2350 Health Sciences Mall, Vancouver, British Columbia, V6T 1Z3, Canada; 4 Canada's Michael Smith Genome Sciences Centre, 570 West 7th Ave - Suite 100, Vancouver, British Columbia, V5Z 4S6, Canada; 5 Bioinformatics Graduate Program, University of British Columbia, 100-570 West 7th Avenue, Vancouver, British Columbia, V5Z 4S6 Canada; 6 Laboratory of Parasitic Diseases, Division of Intramural Research, NIAID, National Institutes of Health, 4 Center Drive, Bethesda, Maryland, 20892, USA; 7 Department of Biochemistry and Molecular Biology, University of British Columbia, Faculty of Science, 2350 Health Sciences Mall, Vancouver, British Columbia, V6T 1Z3, Canada

## Abstract

**Background:**

Leishmania and other intracellular pathogens have evolved strategies that support invasion and persistence within host target cells. In some cases the underlying mechanisms involve the export of virulence factors into the host cell cytosol. Previous work from our laboratory identified one such candidate leishmania effector, namely elongation factor-1α, to be present in conditioned medium of infectious leishmania as well as within macrophage cytosol after infection. To investigate secretion of potential effectors more broadly, we used quantitative mass spectrometry to analyze the protein content of conditioned medium collected from cultures of stationary-phase promastigotes of *Leishmania donovani*, an agent of visceral leishmaniasis.

**Results:**

Analysis of leishmania conditioned medium resulted in the identification of 151 proteins apparently secreted by *L. donovani*. Ratios reflecting the relative amounts of each leishmania protein secreted, as compared to that remaining cell associated, revealed a hierarchy of protein secretion, with some proteins secreted to a greater extent than others. Comparison with an *in silico *approach defining proteins potentially exported along the classic eukaryotic secretion pathway suggested that few leishmania proteins are targeted for export using a classic eukaryotic amino-terminal secretion signal peptide. Unexpectedly, a large majority of known eukaryotic exosomal proteins was detected in leishmania conditioned medium, suggesting a vesicle-based secretion system.

**Conclusion:**

This analysis shows that protein secretion by *L. donovani *is a heterogeneous process that is unlikely to be determined by a classical amino-terminal secretion signal. As an alternative, *L. donovani *appears to use multiple nonclassical secretion pathways, including the release of exosome-like microvesicles.

## Abstract 2

Analysis of Leishmania-conditioned medium resulted in the identification of 151 proteins apparently secreted by the parasitic protozoan Leishmania donovani and suggested a vesicle-based secretion system.

## Background

*Leishmania *spp. are the causative agents of a group of tropical and subtropical infectious diseases termed the leishmaniases. These infections disproportionately affect poorer peoples in developing areas of the world. Because of the debilitating and disfiguring results of infection, these diseases are a great barrier to socioeconomic progress in endemic areas. As of 2001, it was estimated that 12 million people worldwide have been infected with leishmania, and 2 million new cases are believed to occur each year [1]. Recent environmental changes such as urbanization, deforestation, and new irrigation schemes have expanded endemic regions and have led to sharp increases in the number of reported cases [2-4]. In addition, visceral leishmaniasis is establishing itself in previously unaffected areas by piggy-backing on the spread of the HIV epidemic [5]. Leishmania co-infection with HIV has become a serious global health threat. The two infections are involved in a deadly synergy, because leishmania infection exacerbates the immunocompromised state of infected individuals, thereby promoting HIV replication and resulting in earlier onset of AIDS [6]. The combination of HIV co-infection, expansion of endemic regions, and evolving drug resistance [7] has created great need for more effective anti-leishmanial drugs and other control measures. Progress in controlling the leishmaniases requires improved appreciation of the biology of the parasite to allow novel treatment strategies to be designed.

Members of the genus *Leishmania *are digenetic protozoans. The organisms exist either as flagellated, motile promastigotes within the alimentary canal of their phlebotomine sandfly vector or as nonmotile amastigotes that reside within phagolysosomes of mammalian mononuclear phagocytes. Promastigote surface coat constituents have been the focus of considerable interest [8-10], and many of these - including glycoproteins, proteoglycans, and glycolipids - have been shown to play protective roles [8,11,12]. Surface-associated molecules are considered to make up the vast majority of leishmania secreted material [9]. Through these studies, it has become evident that there are a number of unusual features that typify exocytosis by this group of trypanosomatids. For example, in these highly polarized cells, regulated secretion is thought to occur solely at the flagellar pocket, a deep invagination of the plasma membrane from which the single flagellum of leishmania emerges [9,13]. Leishmania are known to synthesize and traffic most surface molecules, such as lipophosphoglycan and leishmanolysin GP63, along the classical endoplasmic reticulum-Golgi apparatus-plasma membrane pathway [9]. As mentioned, these surface molecules are ultimately delivered to the flagellar pocket, and it is thought that the pocket retains its role as the primary if not sole site of secretion in nonflagellated amastigotes [9]. Thus far, no leishmania candidate virulence factors have been shown to traffic through the flagellar pocket. This is not surprising, however, given that no ultrastructural work has accompanied descriptions of leishmania candidate virulence factors, and little attention has been paid to their intracellular or extracellular trafficking pathways.

Whether leishmania use a classical amino-terminal signal sequence peptide to direct the export of most secreted proteins through the flagellar pocket or a different mechanism is unclear. Two leishmania surface glycoproteins, a proteophosphoglycan and GP63, are initially synthesized with a cleavable amino-terminal signal sequence [9]. However, the vast majority of characterized leishmania secreted proteins have no identifiable secretion signal sequence, with the exception of those that are initially membrane bound [9,14,15]. The lack of a clear amino-terminal secretion signal sequence among the majority of characterized leishmania secreted proteins suggests the existence of important nonclassical pathways of secretion.

Despite the potential importance of protein secretion by leishmania, only a small number of leishmania proteins have been examined in detail from this perspective [14,16-18]. Ideally, one would like to know the identities of all of the components of any complex system in order to fully comprehend functionality. Consequently, we set out to identify all, or as many as possible, of the proteins secreted by leishmania. To this end, we designed a quantitative proteomic approach based on SILAC (stable isotopic labeling of amino acids in culture) [19-21]. SILAC involves culturing cells with either normal isotopic abundance amino acids or with stable isotope-enriched amino acids (for instance, L-arginine versus ^13^C_6_-L-arginine) until essentially all proteins of the cell are labeled. The two populations or samples to be compared are then mixed and analyzed by nanoflow liquid chromatography-tandem mass spectrometry (LC-MS/MS). We used this approach to analyze the extent to which any given leishmania protein was secreted into promastigote conditioned medium (Cm) by relating it to the level of the same protein that remained cell associated (CA). In this report, we identified 358 proteins in combined Cm/CA mixtures from *Leishmania donovani *and, based on a quantitative analysis, we conclude that 151 were actively secreted. The general properties of the identified secreted proteins allowed us to postulate potential mechanisms of secretion as well as functional roles within the context of infection.

## Results

### Leishmania conditioned medium contains a multiplicity of enriched proteins

**Figure 1:**
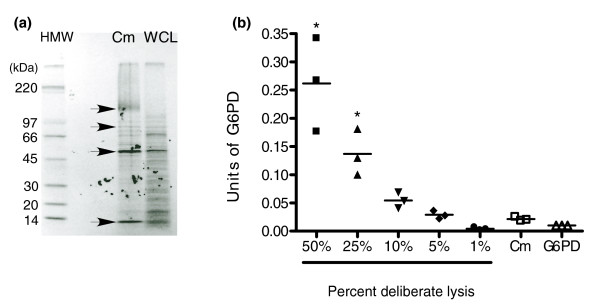
Leishmania Cm contains enriched proteins and is minimally contaminated by incidental cell lysis. **(a) **Leishmania promastigotes were metabolically labeled, as described in Materials and methods. Conditioned medium (Cm) from labeled cells and the cells themselves were collected in parallel, and the proteins present in the Cm and corresponding whole cell lysate (WCL) of promastigotes were precipitated in 10% trichloroacetic acid (TCA). Equal numbers of TCA-precipitated counts/minute of Cm and WCL were fractionated on a 5% to 20% gradient polyacrylamide gel. Arrows indicate proteins specifically enriched in leishmania Cm. The autoradiograph shown is representative of three independent experiments. (HMW) High molecular weight marker. **(b) **To control for inadvertent lysis of organisms during collection of Cm, glucose-6-phosphate dehydrogenase (G6PD) activity in Cm collected from isotopically labeled and nonlabeled cells was measured as described in Materials and methods and compared with the activity associated with deliberately lysed promastigotes. The data shown are the means of measurements from three independent experiments. 0.01 units of G6PD were assayed as a control in each experiment. The asterisks shown indicate a significance difference when compared with Cm (*P *< 0.001), calculated by one-way analysi of variance followed by Bonferroni's correction for multiple comparisons (GraphPad Prism 4.0).

The main objective of this study was to characterize as comprehensively as possible the proteins actively secreted by promastigotes of *L. donovani *into culture medium. Before proceeding with the SILAC and LC-MS/MS analysis, we sought to develop a system in which we were confident that the proteins we were detecting in Cm were not artifacts and were in fact present due to *bona fide *secretion. Previous investigations of protein secretion by leishmania were hampered by the presence of degradation products and by the requirement of the cells for serum [14,22]. In light of these complexities, we included a nontoxic protease inhibitor, soy bean trypsin inhibitor, in the promastigote culture medium during collection and isolation of Cm to minimize degradation of secreted proteins by proteases. Secondly, we reduced Cm collection time to 6 hours or less in order to allow culture of promastigotes under serum-free conditions. Pulse-chase labeling of leishmania with ^35^S-methionine followed by isolation of serum-free Cm showed clearly that leishmania secreted numerous proteins (Figure 1a). Here, an equal number of trichloroacetic acid-precipitated counts/minute of Cm and whole cell lysate (WCL) were analyzed, allowing us to compare directly the intensities of protein bands from Cm and WCL. The results show that some of the leishmania-secreted proteins (arrows in Figure 1a) were clearly enriched in the Cm. It is also important to note that the clearly distinct protein separation patterns of leishmania Cm and WCL indicate that the proteins detected in Cm were unlikely to be artifacts present due to lysis of cells during culture or processing (Figure 1a).

To control further for the possibility of false positive protein detection in Cm caused by inadvertent lysis of promastigotes either spontaneously (due to programmed cell death) or during isolation of Cm, using an enzymatic assay we measured the amount of cytosolic marker glucose 6-phosphate dehydrogenase (G6PD) [23] present in Cm. The total amount of G6PD activity detected in Cm was compared with activities found to be associated with serial dilutions of the total mass of promastigotes that was used to generate the Cm. As shown in Figure 1b, the amount of G6PD detected in Cm never exceeded the total enzyme activity that was associated with 5% of the promastigotes used to generate the Cm. Notably, there was also no difference in the amount of G6PD detected in Cm collected from promastigotes that had been grown in either stable isotope or normal isotopic abundance culture medium (data not shown) during the SILAC analysis described below.

### Quantitative mass spectrometry identifies a wide array of leishmania-secreted proteins

Serum-free leishmania Cm collected from stationary phase promastigotes was fractionated either by one-dimension SDS-PAGE or by in-solution isoelectric focusing and analyzed by LC-MS/MS using a linear trapping quadrupole-Fourier transform hybrid mass spectrometer (see Materials and methods, below). We set three criteria that had to be met for any protein detected by mass spectrometry to be included in the leishmania 'secretome'. First, we only considered proteins to be identified if at least two unique tryptic peptide sequences from that protein were detected (see Materials and methods for peptide criteria limits). Second, we required a particular protein to be observed in at least three out of four independent experiments. This resulted in the identification of 358 proteins (listed in Additional data file 1) in the pooled Cm and CA samples, with an estimated false discovery rate of less than one protein in 200. Interestingly, by these criteria we did not detect G6PD in any of the LC-MS/MS analyses, probably because the amount of G6PD was below the detection limit of the mass spectrometer.

**Figure 2:**
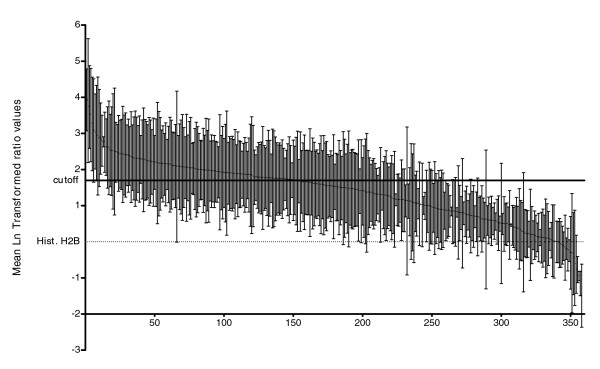
Quantitation of leishmania secreted proteins in Cm. Conditioned medium (Cm)/cell associated (CA) ratios from each of four independent analyses were normalized to the ratio for that of histone H2B followed by log normal (Ln) transformation (see Additional data file 2). Mean values across the four experiments for each protein identity were calculated as described in Materials and Methods. GraphPad Prism 4.0 was then used to calculate the mean and standard deviation (SD) of all Cm/CA values for all proteins found in leishmania Cm (mean = 1.39, SD = 0.85). The same program generated a frequency distribution of the mean Cm/CA values based on the relative frequency of each value within the dataset. Proteins with mean Cm/CA values greater than 2 SDs (1.7; solid black horizontal line) above the value for histone H2B were considered to be members of the secretome. Mean Ln transformed ratios of protein abundance (Cm/CA) where identities lying above the black line are actively secreted, and values falling below the dotted line are likely present due to apoptosis or lysis. X-axis numbers correspond to each protein identity, with 358 in total. The mean and SD from at least three measurements for each protein are shown.

The method of preparation of Cm for LC-MS/MS analysis did not provide sufficient amounts of protein to allow reliable use of standard methods for measuring total protein concentration (see Materials and methods, below), so we estimated the protein content of Cm samples from an initial LC-MS/MS analysis and mixed these with an equal amount of oppositely labeled CA protein. Because this method of equalization is imprecise, we normalized all Cm/CA ratios within an experiment to histone H2B (GeneDB:LmjF19.0050). H2B was consistently detected in Cm, most likely as a result of both general cell lysis and apoptosis [24,25]. After normalization, the values were log_e _transformed (Additional data file 2) and Cm/CA ratios for all identified proteins were calculated as the mean Cm/CA ratio for all peptides from that protein across all experiments (Additional data file 2) [26,27]. These SILAC ratios reflected the degree of enrichment of individual protein species in leishmania Cm, and a frequency distribution is shown in Figure 2. Across all experiments the overall mean ± standard deviation Cm/CA value for the 358 proteins was 1.35 ± 0.85 (Figure 2).

We used the Cm/CA ratio of histone H2B to define the third criterion for inclusion in the secretome. We considered leishmania proteins with a mean Cm/CA peptide ratio at least two standard deviations (1.7) above the ratio for histone H2B (after transformation = 0) to be actively secreted by leishmania (Figure 2, solid line). In choosing this rather conservative yet arbitrary cut-off, we reasoned that if H2B was representative of proteins externalized by apoptosis then, by allowing a significant margin of error around it, the proteins (numbering 151 in total) with Cm/CA ratios of 1.7 or greater were likely to be *bona fide *secreted proteins. This conservative approach provided a high level of specificity for 'secretion' at the expense of sensitivity.

**Figure 3:**
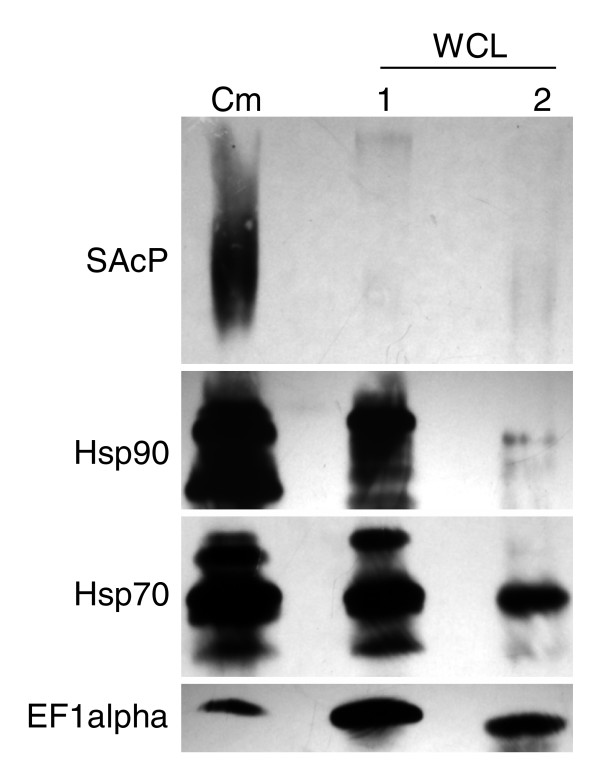
Leishmania HSPs are enriched in Cm. Leishmania conditioned medium (Cm) was collected from a culture containing about 2 × 10^9 ^promastigotes. The proteins contained therein were precipitated and then solubilized directly in Laemmli sample buffer. One half of this volume (containing a known amount of protein) was loaded into the lane labeled 'Cm'. From the 2 × 10^9 ^promastigotes recovered from the culture, 5% (1 × 10^8^) were removed and processed in parallel with the remaining 95%. The 5% figure was chosen based upon the estimated (see Figure 1) maximum number of organisms that may have undergone incidental lysis during the incubation period and the collection centrifugation. Proteins contained in the whole cell lysate (WCL) prepared from the 1 × 10^8 ^cells were precipitated and resolubilized in a volume equal to that used to resolubilize the proteins precipitated from Cm collection. To allow for direct comparison to be made, half of this volume was then loaded into WCL lane #1. Protein was also precipitated from the WCL prepared from the remaining 95% of the cells and after solubilization in sample buffer an amount of protein was loaded into WCL lane #2 equal to that loaded into the lane labeled Cm. After transfer to nitrocellulose membrane, blots were probed, stripped, and reprobed with the indicated antibodies. The data shown are representative of results obtained in at least three identical experiments. EF1alpha, elongation factor-1α; Hsp, heat shock protein; SAcP, secreted acid phosphatase.

We used Western blotting to examine a select group of proteins in paired Cm and CA samples to determine the extent to which this orthogonal method of detection would correlate with the SILAC/mass spectrometry analysis. Here we examined four proteins: heat shock protein (HSP)70, with a Cm/CA value of 1.86, above the cut-off of histone H2B plus two standard deviations (or +1.70); HSP83/HSP90, with a Cm/CA ratio of 1.50 falling just below the cut-off; elongation factor-1α (EF-1α) with a ratio of 0.69; and secreted acid phosphatase (SacP), which was not detected by LC-MS/MS. As shown in Figure 3, SAcP was detected as a dispersed band in Cm, but it was completely absent from the aliquots of WCL analyzed (lanes 1 and 2). Both HSP70 and HSP90 were also clearly enriched in Cm, with HSP70 to a greater extent than HSP90 (compare Cm with WCL lane 2). On the other hand, the bulk of EF-1α was retained intracellularly (Figure 3). This qualitative analysis indicated that the SILAC/LC-MS/MS results correlated closely with conventional protein detection by Western blotting in respect of providing a semiquantitative estimate of protein secretion by leishmania. Additionally, these findings indicated that the arbitrary third criterion for inclusion in the secretome was both valid and in fact highly rigorous, because HSP90 - a protein falling just below the secretome cut-off (Cm/CA of 1.7; Figure 2) - was clearly found to be enriched in Cm by Western blotting (again compare Cm with WCL lane 2).

The results for SAcP both by mass spectrometry and Western blotting were of particular interest and appeared to be a special case. Whereas this ecto-enzyme, which was previously reported to have an amino-terminal secretion signal [28], was highly enriched in Cm (Figure 3), its absence from the LC-MS/MS analysis suggested that its absolute abundance must be quite low. This is addressed further under Discussion (below).

The results of the Western blotting also indicated that there was minimal contamination of Cm by incidental lysis. Figure 3 shows the protein profile of 5% of the cells (selected based on the maximum amount of lysis that may have occurred according to the results of the G6PD analysis; Figure 1b) to be markedly distinct from that of the leishmania Cm (compare Cm with WCL lane 1). The distinct profiles of Cm and WCL observed in the metabolic labeling experiment (Figure 1a) also indicated that contamination of Cm through lysis was negligible.

### Gene Ontology analysis of the leishmania secretome

To develop an understanding of how protein secretion by leishmania might be related to specialized functions or processes, we used the *Leishmania *Genome [29] and the Gene Ontology (GO) [30] databases in conjunction with the Blast2GO analysis tool [31] to determine whether any classes of proteins were more likely to be found in among the leishmania secreted proteins. This analysis resulted in 85% of the proteins detected in leishmania Cm having one or more GO term assignments (Additional data file 3).

**Figure 4:**
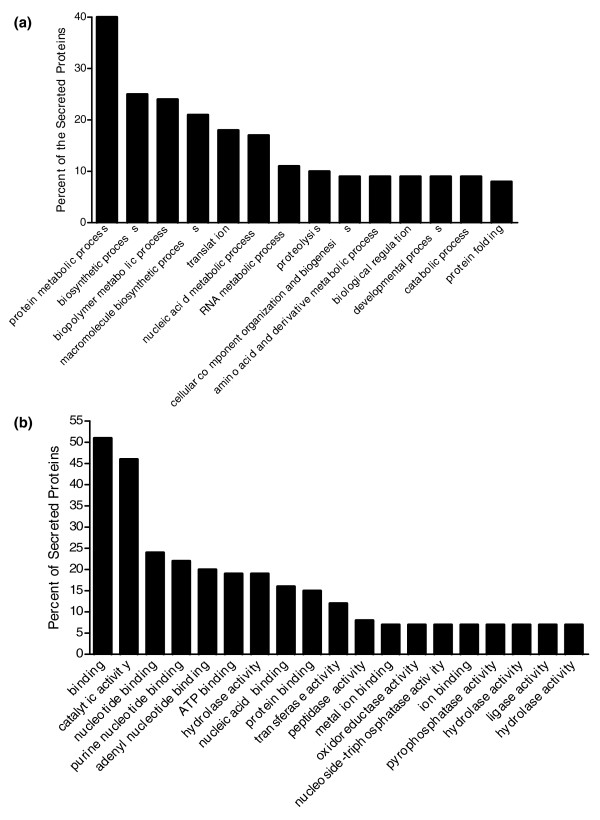
High prevalence GO assignments in the leishmania secretome. The secretome sequences were categorized according to **(a) **biological process and **(b) **molecular function. Nonredundant processes and functions assigned to at least ten leishmania-secreted protein sequences are displayed. Bars indicate the number of protein sequences found under each Gene Ontology (GO) term expressed as a percentage of the total 151 actively secreted proteins.

After tallying the number of leishmania secreted proteins assigned to each GO term, it was clear that many of the secreted proteins (Figure 4a) were involved in turnover and synthesis of protein and nonprotein macromolecules. In fact, 27 out of the 151 secreted proteins (18%) were predicted to be involved in protein translation (GO: 0006412), which was more than in any other discrete biologic process (Figure 4a). Beyond this, as shown in Figure 4a, the leishmania secreted proteins identified by LC-MS/MS were found to be involved in a wide array of processes, including proteolysis (GO:0006508), protein folding (GO:0006457), and biologic regulation (GO:0065007).

Consistent with the biological process GO analysis, a full 50% of leishmania secreted proteins were involved in protein binding interactions, for example binding to ATP (GO:0005524), ions (GO:0043167), or other proteins (GO:0005515; Figure 4b). Other highly represented functions included pyrophosphatase activity, hydrolase activity, and oxidoreductase activity (GO:0016462, GO:0016787, and GO:0016491). It is noteworthy that nearly 20 proteins that fell below the secretion cut-off were annotated as having transporter activity (GO:0005215), whereas no such activity was found for the secreted proteins (Additional data file 3).

**Figure 5:**
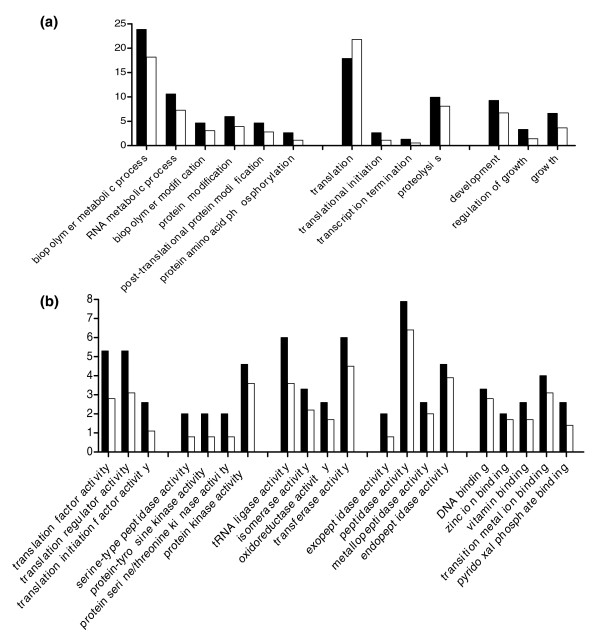
GO assignments concentrated in the leishmania secretome. **(a) **Biological process and **(b) **molecular function Gene Ontology (GO) terms with an equivalent or greater number of assignments in the secreted proteins (black bars) compared with all of the conditioned medium (Cm) proteins (white bars) are shown. Bars indicate the number of actively secreted protein sequences found under each GO term expressed as a percentage of the 151 actively secreted proteins (black bars) compared with the total number of Cm sequences found under that GO term expressed as a percentage of the total 358 proteins identified in the Cm (white bars).

Of interest, there appeared to be a trend toward concentration of a distinct set of processes and functions in the group of 151 leishmania proteins making up the leishmania secretome. As shown in Figure 5a, when compared with the total group of 358 proteins consistently identified in Cm, there appeared to be enrichment of proteins involved in processes related to growth (GO:0040007), RNA metabolism (GO:0016070), and biopolymer modification (GO:0043412), including protein amino acid phosphorylation (GO:0006468). Consistent with these biologic process assignments, molecular functions such as kinase activity, peptidase activity, and translation factor activity (GO:0016301, GO:0008233, and GO:0003746, respectively) appeared to be more prevalent among the group of 151 leishmania secreted proteins than among the total group of 358 proteins consistently identified in Cm (Figure 5b). We used the GOSSIP [32] statistical framework to determine whether any GO terms were significantly enriched in the secreted proteins when compared to other Cm proteins. Many of the processes and functions discussed and depicted in Figure 5 had significant (*P *< 0.05) single test *P *values. However, after correcting for multiple testing using both a false discovery rate (the most common correction method) and a family wise error rate (which is more correct in this context because there was no *a priori *basis for an association between the secreted proteins and any GO term) [32], no terms were found to be significantly enriched in the group of 151 secreted proteins. This may be due to our small sample size of individual GO terms associated with at most 358 proteins. In contrast, these statistical tests are regularly carried out on sample sizes in the tens of thousands [32] of genes or proteins. In addition, statistical significance may not have been achieved because we were comparing two datasets with a high probability of overlap, because we looked for enrichment of GO terms associated with the group of 151 proteins in leishmania secretome compared with GO terms associated with the total group of 358 Cm proteins. In fact, some of the Cm proteins below the cut-off may be actively secreted and certainly were found to be exported by some mechanism, including cell death. For these reasons, we consider that the apparent concentration of GO associations shown in Figure 5 may in fact be meaningful.

In addition to members of the secretome having pleiotropic functions, they were also predicted to have a variety of subcellular localizations. Nearly one-third of leishmania secreted proteins were predicted to be cytoplasmic (GO:0005737) by GO, and these had associations with both membrane bound (GO:0043227) and nonbound intracellular organelles (GO:0043228), including ribosomal proteins, nuclear proteins, mitochondrial proteins, and glycosomal proteins (Additional data file 3). Only five secreted proteins were predicted to be integral membrane proteins, and none of the secretome proteins were predicted to be associated with the endoplasmic reticulum.

### Bioinformatics analysis of secreted proteins in the leishmania genome

We screened the leishmania genome database for proteins containing a classical amino-terminal secretion signal peptide, in order to generate a putative list of classically secreted proteins for comparison with the proteins identified by LC-MS/MS. We modified a bioinformatics approach previously used to identify proteins secreted by *Mycobacterium tuberculosis *[33] and applied it to the genome of *Leishmania major *[34]. Proteins were considered highly likely to be secreted if the sequence included a classical amino-terminal secretion signal peptide and lacked additional transmembrane (TM) domains. Additional TM domains would have suggested that the protein was membrane bound and therefore unlikely to be released from the cell. The majority of leishmania surface expressed proteins are associated with the plasma membrane via a glycophosphotidylinositol (GPI) lipid attachment [9], and some of these GPI-attached surface proteins, such as GP63, are known to disassociate from the membrane and can be detected in Cm [35]. In light of this, as a final step we screened the proteins positive for a signal sequence and negative for multiple TM domains for GPI-linkage attachment sites and considered positive proteins to be secreted (Additional data file 4). Using these parameters, we found that the leishmania genome encodes 217 proteins that contain a classical secretion signal peptide, of which 141 are annotated as hypothetical proteins (Additional data file 4). Of the remaining 76 proteins, approximately one-third appear to be gene duplications, leaving 50 unique leishmania proteins with a known or putative classical eukaryotic secretion signal peptide. It is of interest that only one of the proteins we predicted to be secreted via an amino-terminal secretion signal - LmjF16.0790, a chitinase - has previously been demonstrated to be secreted by leishmania promastigotes [16,36], although we did not detect this protein in our LC-MS/MS analysis.

Our analysis also suggests that SAcP does not contain a classical secretion signal, contrary to a previous report [37]. Based upon the Von Heijne algorithm [34], the latter study predicted the presence of a 23-amino-acid amino-terminal 'signal peptide'. Subsequently, this leader peptide was shown to be sufficient for secretion of a green fluorescent protein fusion construct expressed in *L. donovani *[27]. The SignalP algorithm we used is the updated version of the 1985 Von Heijne algorithm. The lack of concordance in these predictions highlights the limitations of bioinformatics, while reinforcing the well known fact that signal sequences are highly variable.

Our bioinformatics analysis also confirmed the annotation in the *Leishmania *Genome database [29] that none of the histidine secretory acid phosphatases found in the genomes of *L. major *or *L. donovani infantum *have classical amino-terminal secretion signals. Interestingly, only the membrane bound acid phosphatases of *L. major *are annotated as containing classical secretion signal peptides, whereas the same is not true of the orthologs in *L. donovani infantum*, and these membrane bound proteins would have been excluded by our TM domain screen. Only 14 of the proteins predicted to be secreted through a classical signal sequence-dependent mechanism were detected in leishmania Cm by MS, and only two of these, GeneDB:LmjF04.0310 and LmjF36.3880, had sufficiently high SILAC ratios to be included in the secretome (Additional data file 4). Although there are several possible explanations for failing to detect a protein by LC-MS/MS, the lack of correlation between the measured and the *in silico *predicted secretomes suggests that leishmania utilize nonclassical secretion signals and pathways to regulate the export of the majority of secreted proteins.

### Evidence that proteins released by leishmania may originate in exosome-like vesicles, apoptotic vesicles, and glycosomes

**Figure 6:**
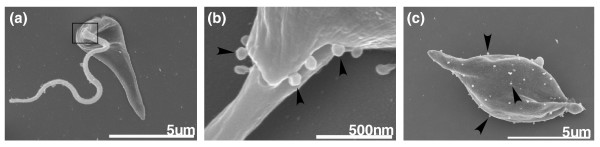
Microvesicles budding from the flagellar pocket and plasma membrane of leishmania. Stationary phase leishmania promastigotes were fixed and coated for scanning electron microscopy as described in Materials and methods. **(a) **A leishmania promastigote, **(b) **10× magnification of the exposed flagellar pocket region of panel a (square) after stage rotation, and **(c) **a promastigote in the process of differentiating into an amastigote. Arrowheads point to microvesicles.

Somewhat unexpected was the finding that leishmania Cm contained all of the proteins identified previously to be associated with exosomes isolated from both B lymphocytes and dendritic cells, with the exception of those for which the leishmania genome does not contain an ortholog (Additional data file 5). In fact, more than 10% of the proteins found in the leishmania secretome were previously detected in exosome-like microvesicles released from other eukaryotic cells (Table 1), including B lymphocytes [38], dendritic cells [24], and adipocytes [39]. Recently, mammalian adipocytes were shown to secrete microvesicles, which were referred to as adiposomes [39]. These adiposomes contained 98 proteins, 13 of which we concluded to be actively secreted (Table 1). At least 25 additional adiposome proteins were detected in leishmania Cm with relative abundances lower than the secretome cut-off (Additional data file 5). The concordance of the proteomic data between these higher eukaryotic secreted microvesicles and the leishmania secretome is remarkable. These findings suggested that leishmania secrete exosome/adiposome-like microvesicles carrying proteomic cargo that is similar in composition to host microvesicles. In support of this, using scanning electron microscopy, we observed 50 nm microvesicles specifically located at the mouth of the leishmania promastigote flagellar pocket (Figure 6a,b), as well as evenly distributed across the cell surface of cells with the apparent morphology of amastigotes undergoing differentiation axenically (Figure 6c).

**Table 1:** Leishmania-secreted proteins associated with exosome-like and glycosomal vesicles

GeneDB accession number	Protein identification^a^	Mean Cm/CA ratio	Microvesicle association
LmjF35.3340	6-Phosphogluconate dehydrogenase, decarboxylating, putative	3.01	GLY
LmjF29.0510	Cofilin-like protein	2.80	DC
LmjF36.6910	Chaperonin, putative, T-complex protein 1 (theta subunit), putative	2.61	AP
LmjF01.0770	Eukaryotic initiation factor 4a, putative	2.60	DC
LmjF28.2860	Cytosolic malate dehydrogenase, putative	2.20	AP
LmjF24.2060	Transketolase, putative	2.19	GLY
LmjF33.2550	Isocitrate dehydrogenase, putative	2.16	AP
LmjF28.2770	Heat-shock protein hsp70, putative	2.14	BC, DC, AP
LmjF35.3860	T-complex protein 1, eta subunit, putative	2.14	AP
LmjF12.0250	Cysteinyl-tRNA synthetase, putative	2.07	GLY
LmjF14.1160	Enolase	2.05	BC, DC, AP
LmjF36.2030	Chaperonin Hsp60, mitochondrial precursor	2.03	AP
LmjF23.1220	T-complex protein 1, gamma subunit, putative	2.00	AP
LmjF05.0350	Trypanothione reductase	1.99	GLY
LmjF36.2020	Chaperonin Hsp60, mitochondrial precursor	1.98	AP
LmjF36.1630	Clathrin heavy chain, putative	1.98	BC, AP
LmjF16.0540	Aspartate carbamoyltransferase, putative	1.95	GLY
LmjF27.2000	Hypothetical protein, conserved	1.90	GLY
LmjF31.1070	Biotin/lipoate protein ligase-like protein	1.89	AP
LmjF26.1240	Heat shock protein 70-related protein	1.86	BC, DC, AP
LmjF04.0960	Adenylate kinase, putative	1.77	GLY
LmjF27.1260	T-complex protein 1, beta subunit, putative	1.76	AP
LmjF30.3240	Glutamyl-tRNA synthetase, putative	1.74	GLY
LmjF21.0810	Methionyl-tRNA synthetase, putative	1.74	GLY
LmjF36.3210	14-3-3 Protein-like protein	1.74	DC, AP
LmjF33.2540	Carboxypeptidase, putative, metallo-peptidase	1.73	GLY

^a ^The Mascot algorithm {2008 609/id} was used to identify the protein names and the GeneDB accession numbers [29]. Microvesicles: BC, B-cell lymphocyte exosome [38,62]; DC, dendritic cell exosome [24]; AP, adipocyte exosome (adiposome) [39]; and GLY, leishmania glycosome [42]. CA, cell associated; Cm, conditioned medium; Hsp, heat shock protein.

Surprisingly, DNA-binding histone proteins were reliably detected by LC-MS/MS in Cm of stationary phase promastigotes (Additional data files 1 and 5). Histone proteins have been detected in dendritic cell exosomal preparations and were shown to enrich in these preparations after the cells were treated with an apoptosis-inducing agent [24]. The dendritic cell vesicles containing histone proteins were more electron dense and migrated to a slightly higher sucrose density than the exosomes [24]. This led the authors to conclude that the histone-containing vesicles were indeed a distinct population of vesicles, termed apoptotic vesicles or blebs [24]. The detection of histones in Cm of stationary phase leishmania (Additional data files 1 and 5), along with the significant number of apoptotic leishmania known to be present in a stationary phase population (approximately 43 ± 5%) [25], suggests that promastigotes may have been releasing apoptotic vesicles as well as exosomes.

In addition to exosomal and apoptotic vesicle-associated proteins, we also found that the leishmania secretome included many of the major glycolytic enzymes that normally reside in glycosomes of kinetoplastid organisms [40] (Table 1 and Additional data file 5). Relevant to these findings, leishmania have been shown to utilize peroxisomal targeting signals (PTSs; PTS1 and PTS2) to direct proteins to the glycosome [41], and a screen of the leishmania genome identified approximately 100 proteins with either a PTS1 or a PTS2 targeting signal [42]. Remarkably, our MS analysis of leishmania Cm detected nearly half of these predicted glycosomal proteins, with ten being detected at high enough relative abundance to be considered *bona fide *secreted proteins (Table 1). These findings suggest that leishmania release either whole glycosomes or glycososomal cargo into the extracellular environment.

## Discussion

To our knowledge, this report is the first proteomic analysis of protein secretion by *Leishmania*, or, for that matter, any other kinetoplast. This quantitative proteomic analysis showed that *L. donovani *released a wide array of proteins when in the stationary phase of growth (Additional data file 1). Based on previous studies concerned with the pathogenesis of leishmania as well as other intracellular pathogens [17,43], we anticipated that leishmania may secrete virulence effectors into their extracellular environment, including the cytosolic compartment of infected host cells. By examining the composition of the leishmania secretome and generating quantitative information concerning the relative enrichment of secreted proteins, we expected to identify candidate leishmania effector proteins that may be involved in virulence. As expected, protein export was found to be heterogeneous, with some proteins exported to a higher degree than they were retained by the cell, whereas for others the opposite was true (Figure 2). It was our assumption that proteins with higher Cm/CA ratios were more likely to be actively secreted than they were to be externalized as a result of either incidental lysis or apoptosis. In light of this, we used the relative abundance data and a rigorous statistical cut-off (Cm/CA values greater than the ratio for H2B by at least two standard deviations) to define proteins actively secreted by leishmania. Based on this analysis, we consider 151 proteins in this dataset to be *bona fide *members of leishmania secretome. On the other hand, we recognize that in implementing this rigorous cut-off we probably sacrificed some sensitivity. Thus, it is probable that at least some proteins with ratios falling below the cut-off are actively secreted as well.

Next, we inspected the leishmania secretome for potential virulence factors. Candidate virulence factors were divided into four categories: proteins putatively involved in intracellular survival; proteins with known immunosuppressive functions; proteins involved in signal transduction; and proteins involved with transport processes (Table 2). By far the largest class of candidate virulence factors was comprised of proteins that may be required for intracellular survival. The leishmania secretome showed a remarkable abundance of proteasome subunits (such as GeneDB:LmjF35.4850, LmjF36.1600, LmjF21.1700, LmjF21.1830, LmjF27.0190, LmjF36.1650, and LmjF34.0650) and proteases such as the oligopeptidases (GeneDB:LmjF09.0770; Tables 2 and 3, and Additional data file 1), of which many had high Cm/CA values. In addition, proteolysis was one of the most common GO terms assigned to the leishmania secreted proteins. Although the frequency of this term did not reach statistical significance (see Gene Ontology analysis of the leishmania secretome, under Results, above), this term appeared to be somewhat over-represented among the proteins in the upper half of the ratio distribution (Figures 4a and 5a). It seems likely that the secretion of at least some of these proteins may be part of a stress response. On the other hand, some of these proteins may be involved in pathogenesis. One potential mechanism is the direction of their proteolytic activities toward degradative enzymes resident in phagolysosomes to promote intracellular survival. A second possibility might involve direction of their proteolytic activities to degrade major histocompatibility complex class I and II molecules, thereby preventing antigen loading and reduced efficiency of antigen presentation, as has been described for leishmania-infected cells [44]. These findings suggest that secreted leishmania proteins with proteolytic activities may contribute to pathogenesis, and further investigation of this is warranted.

**Table 2:** Leishmania candidate virulence factors enriched in Cm

GeneDB accession numbers	Protein identification^a^	Mean Cm/CA ratio^b^	Putative function^c^
Signal transduction proteins			

LmjF35.2420	Phosphoinositide-binding protein, putative	3.51	Kinase^d^
LmjF33.1380	Mitogen-activated protein kinase 11, putative, MAP kinase, putative	2.42	Kinase
LmjF09.0770	Oligopeptidase b, serine peptidase, clan SC, family S9A-like protein	2.31	Cell-cell signaling
LmjF34.2820	Regulatory subunit of protein kinase a-like protein	2.30	Kinase
LmjF28.2740	Activated protein kinase c receptor (LACK)	2.28	Kinase receptor
LmjF25.0750	Protein phosphatase, putative	2.27	Phosphatase
LmjF35.1010	Casein kinase, putative	2.08	Kinase
LmjF10.0490	Mitogen-activated protein kinase 3, putative, MAP kinase 3, putative	1.73	Kinase, signal transduction
LmjF31.2790	ADP-ribosylation factor, putative	1.67	Small GTPase mediated signal transduction

Immunosupressive proteins			

LmjF35.2210	Kinetoplastid membrane protein-11	2.33	Immunosuppressive [51]
LmjF33.1750	Macrophage migration inhibitory factor-like protein	2.21	Immunosuppressive [52]
LmjF25.0910	Cyclophilin a	1.73	Immunosuppressive [103]

Proteins Involved in Intracellular survival			

LmjF14.1360	Myo-inositol-1-phosphate synthase	3.93	Inositol biosynthesis [50]
LmjF23.0200	Endoribonuclease L-PSP (pb5), putative	3.91	mRNA salvage, inhibition protein synthesis
LmjF11.0630	Aminopeptidase, putative, metallo-peptidase, clan MF, family M17	3.05	Proteolysis
LmjF28.1730	Proteasome regulatory non-ATP-ase subunit 2, putative	2.40	Proteolysis
LmjF34.1040	Uracil phosphoribosyltransferase, putative	2.34	Pyrimidine salvage
LmjF09.0770	Oligopeptidase b, serine peptidase, clan SC, family S9A-like protein	2.31	Invasion, proteolysis
LmjF32.1820	Iron superoxide dismutase, putative	2.29	Antioxidant
LmjF13.0090	Carboxypeptidase, putative, metallo-peptidase, clan MA(E), family 32	2.14	Proteolysis
LmjF28.2770	Heat-shock protein hsp70, putative	2.14	Protein stability
LmjF26.1570	Thimet oligopeptidase, putative, metallo-peptidase, clan MA(E), family M3	2.10	Proteolysis
LmjF05.0960	Dipeptidyl-peptidase III, putative, metallo-peptidase, clan M-, family M49	2.08	Proteolysis
LmjF14.1160	Enolase	2.05	Plasminogen binding [104], Invasion
LmjF19.0160	Aminopeptidase, putative, metallo-peptidase, clan MG, family M24	2.04	Proteolysis
LmjF21.1830	Proteasome alpha 5 subunit, putative	2.03	Proteolysis
LmjF05.0350	Trypanothione reductase	1.99	Antioxidant
LmjF21.0760	Proteasome regulatory non-ATP-ase subunit 5, putative,19S proteasome regulatory subunit	1.97	Proteolysis
LmjF23.0270	Pteridine reductase 1	1.92	Antioxidant
LmjF26.0810	Glutathione peroxidase-like protein, putative	1.89	Antioxidant
LmjF27.0190	Proteasome alpha 7 subunit, putative	1.89	Proteolysis
LmjF26.2280	Nitrilase, putative	1.86	Carbon-nitrogen hydrolase
LmjF26.1240	Heat shock protein 70-related protein	1.86	Protein stability
LmjF31.1890	Peptidase m20/m25/m40 family-like protein	1.85	Proteolysis
LmjF06.0140	Proteasome beta 6 subunit, putative,20S proteasome beta 6 subunit, putative	1.85	Proteolysis
LmjF29.0120	Proteasome regulatory non-ATPase subunit, putative	1.81	Proteolysis
LmjF34.0650	Proteasome regulatory non-ATP-ase subunit 11, putative,19S proteasome regulatory subunit, metallo-peptidase, Clan MP, Family M67	1.79	Proteolysis
LmjF35.0750	Proteasome activator protein pa26, putative	1.78	Proteolysis
LmjF35.2350	Aminopeptidase P, putative, metallo-peptidase, clan MG, family M24	1.77	Proteolysis
LmjF02.0370	Proteasome regulatory non-ATPase subunit 6, putative	1.77	Proteolysis
LmjF36.3210	14-3-3 protein-like protein	1.74	Anti-apoptotic
LmjF21.1700	Proteasome alpha 2 subunit, putative	1.66	Proteolysis
LmjF36.1600	Proteasome alpha 1 subunit, putative	1.64	Proteolysis
LmjF35.4850	Proteasome alpha 1 subunit, putative	1.60	Proteolysis

Proteins involved in vesicular transport processes			

LmjF35.2420	Phosphoinositide-binding protein, putative (sorting nexin 4)	3.51	Transport of proteins and other substances
LmjF27.0760	Small GTP-binding protein Rab1, putative	2.27	Endosomes/Golgi trafficking
LmjF32.1730	Coatomer epsilon subunit, putative	2.09	Intracellular protein transport
LmjF36.1630	Clathrin heavy chain, putative	1.98	Endocytosis, trans-Golgi to lysosome trafficking
LmjF18.0700	Hypothetical protein, conserved	1.85	HEAT repeat, intracellular protein transport
LmjF31.2790	ADP-ribosylation factor, putative	1.67	Intracellular protein transport

^a^The Mascot algorithm {2008 609/id} was used to identify the protein names and the GeneDB accession numbers [29]. ^b^Mean of normalized, log normal (Ln) transformed conditioned medium (Cm)/cell associated (CA) peptide ratios for at least three of four experiments. ^c^Putative functions and locations are derived from the GeneDB database {2007 238/id} unless otherwise noted. ^d^Protein sequence contains an amino-terminal secretion signal peptide according to SignalP.

**Table 3:** Highly enriched leishmania secreted proteins

GeneDB accession number	Protein identification^a^	Mean Cm/CA ratio^b^	Function^c^	Predicted location^c^
LmjF14.1360	Myo-inositol-1-phosphate synthase	3.93	Inositol biosynthesis [50]	Cytosol
LmjF23.0200	Endoribonuclease L-PSP (pb5), putative	3.91	Nuclease, mRNA cleavage	Cytosol
LmjF15.1203	60S acidic ribosomal protein P2	3.73	Translation [102]	Ribosome^d^
LmjF35.2420	Phosphoinositide-binding protein, putative	3.51	Phosphoinositol binding, signal transduction	Cytosol^d^
LmjF16.0140	Eukaryotic translation initiation factor 1A, putative	3.26	Translation	Cytosol, exosomes [24,38,39]
LmjF32.2180	Hypothetical protein, conserved	3.08	Translation initiation	Nucleus
LmjF11.0630	Aminopeptidase, putative, metallo-peptidase, Clan MF, Family M17	3.05	Proteolysis	Cytosol
LmjF35.3340	6-Phosphogluconate dehydrogenase, decarboxylating, putative	3.01	Glucose cataboloism	Cytosol
LmjF04.0310	Beta-fructofuranosidase, putative	2.93	Carbohydrate metabolism	Cytosol^d^
LmjF36.3840	Glycyl tRNA synthetase, putative	2.87	Translation	Cytosol

^a^The Mascot algorithm {2008 609/id} was used to identify the protein names and the GeneDB accession numberes [29]. ^b^Mean of normalized, log normal (Ln) transformed conditioned medium (Cm)/cell associated (CA) peptide ratios for at least three of four experiments. ^c^Putative functions and locations are derived from the GeneDB database {2007 238/id}unless otherwise noted. *Protein sequence contains an amino-terminal secretion signal peptide according to SignalP.

Also likely to be involved in intracellular survival are secreted antioxidants, and more generally proteins with oxidoreductase activity, such as iron superoxide dismutase (GeneDB:LmjF32.1820). Other examples of these were found in the leishmania secretome (Figure 5b and Additional data file 1), and these may provide protection from intracellular free radical attack. In addition, some members of the secretome, such as the putative 14-3-3 protein, are known to have powerful antiapoptotic properties in other systems [45]. That leishmania infection inhibits host cell apoptosis is well known [46,47], and these antiapoptotic secreted proteins may be active in prolonging the lifespan of infected host cells.

An important inclusion to the category of proteins with functional roles in intracellular survival were nucleases, such as GeneDB:LmjF23.0200, an endoribonuclease, which was found to be the second most highly secreted protein (Table 3). This endoribonuclease belongs to a class of proteins that act on single-stranded mRNA and are thought to be inhibitors of protein synthesis [48]. These nucleases may aid in purine salvage, which is obligatory for leishmania because they are incapable of *de novo *purine synthesis [49].

Myo-inositol-1-phosphate synthase (GeneDB:LmjF14.1360), the protein with the highest relative abundance ratio (Table 3) and therefore the most enriched in the Cm, may also play a role in intracellular survival (Table 2). Leishmania myo-inositol-1-phosphate synthase has been shown to be essential for growth and survival in myo-inositol limited environments [50]. Leishmania myo-inositol-1-phosphate synthase knockouts were found to be completely avirulent [50] in mice, suggesting that the phagolysosomal lumen may be a myo-inositol limited environment. Myo-inositol-1-phosphate synthase is required for *de novo *biosynthesis of myo-inositol, a precursor of vital inositol phospholipids such as those found in the GPI membrane anchors of nearly all leishmania surface proteins and other glycoconjugates such as GP63 and lipophosphoglycan. The massive export of this essential enzyme into Cm is intriguing and warrants further study.

The leishmania secreted protein kinetoplastid membrane protein-11 (GeneDB:LmjF35.2210), identified in the SILAC/mass spectrometry analysis, was previously characterized as having immunomodulatory effects on host cells during leishmania infection [51]. Furthermore, we found that the leishmania secretome contains an ortholog of the mammalian macrophage migration inhibitory factor (GeneDB:LmjF33.1750), a protein with known immunosuppressive and immunomodulatory properties [52] in humans. It is possible that this leishmania ortholog could share these functions and affect host immune responses during leishmania infection.

Manipulation of host cell function via interference with signaling pathways is a well known virulence tactic of intracellular pathogens [53-57]. After internalization, leishmania infected macrophages exhibit defective signaling in response to various stimuli [54,55,57]. Based on our analysis, we estimate that at least ten secreted leishmania proteins are predicted to be involved in some manner in signal transduction (Table 2 and Additional data file 3). In this regard, we found that kinase activity was concentrated in the upper half of the secretome ratio distribution (Figure 5b). Secreted leishmania signaling intermediates such as the mitogen-activated protein kinases 3 and 11 (GeneDB:LmjF33.1380 and LmjF10.0490) and the protein tyrosine phosphatase-like protein (GeneDB:LmjF16.0230) have the potential to affect macrophage cell signaling after internalization [56]. Another interesting signaling related protein, the putative phosphoinositide-binding protein (GeneDB:LmjF35.2420), was one of most highly secreted proteins (Table 3). This protein might influence macrophage cell signaling through its potential binding of inositol containing signaling intermediates that are products of phosphatidyloinositol 3 kinase. Notably, GO analysis identified this putative phosphoinositide-binding protein (GeneDB:LmjF35.2420) as a sorting nexin 4-like protein (Additional data file 3). Sorting nexins are known to be involved in coordinating intracellular vesicle trafficking processes, including both endocytosis and exocytosis [58]. As such, this putative sorting nexin may be considered to be a leishmania candidate virulence factor for its potential to modulate vesicle trafficking in infected cells (Table 2).

Somewhat unexpected was the finding of proteins in leishmania Cm known to be involved in vesicular transport (Tables 2 and 1), such as the phosphoinositide binding protein discussed above, the small GTP-binding protein Rab1 (GeneDB:LmjF27.0760) and a putative ADP-ribosylation factor (GeneDB:LmjF31.2790). We have classified these proteins as candidate virulence factors because, although these transport vesicle regulatory proteins may normally regulate vesicle trafficking in leishmania, ectopically following secretion, they may have the potential to affect vesicle trafficking in infected cells. For example, it is tempting to speculate that these leishmania secreted proteins could directly affect phagosome maturation through modulating transport to and fusion with host multivesicular bodies, endosomes, and lysosomes.

Another interesting and unexpected aspect of the leishmania secretome was the presence of numerous proteins related to translational machinery (Figure 4a and Additional data file 3). The functional basis for this is unclear at this time. Perhaps the turnover of these proteins is extremely high and excess machinery is disposed of via secretion in addition to the reported processes of ubiquitination and proteasome mediated degradation. Interestingly, clathrin-coated vesicles isolated from rat liver [59] were found to contain more than 30 of the same translation related proteins we found in leishmania Cm, including a putative leishmania eukaryotic translation initiation factor 1A (GeneDB:LmjF16.0140), the protein with the fifth highest enrichment ratio (Table 3). Appreciation of the multifunctional nature of proteins is increasing, and the possibility exists that these proteins, perhaps purposely packaged in leishmania secretory vesicles, may play ancillary roles in pathogenesis or pathogen survival, similar to what appears to be the case for EF-1α [17].

The protein secretion pathways utilized by leishmania are not well understood. According to our analysis, only two of the 151 proteins in the leishmania secretome contain a classical amino-terminal secretion signal (Additional data files 2 and 4). The fact that more than 98% of the secretome lacks a targeting signal indicates that nonclassical secretion pathways are probably the dominant means by which leishmania proteins are secreted. In support of this argument, the leishmania secretome included a large number of proteins previously identified as components of exosomes secreted from various higher eukaryotic cell types (Table 1). Leishmania Cm also contained many proteins shown to be cargo of clathrin-coated vesicles. Rat liver clathrin-coated vesicles were found to contain a total of 346 proteins, and in addition to the 30 translation-related proteins mentioned above, an further 30 of these proteins were detected in leishmania Cm, including clathrin (GeneDB:LmjF36.1630) and HSP70. Significantly, both clathrin and HSP70 have been found in exosomes released from various human cells [24,38,39]. In fact, the proteomes of these clathrin-coated vesicles and that of mammalian exosomes were strikingly similar [24,59-62]. Furthermore, leishmania have been shown to form clathrin-coated vesicles [63], and clathrin-directed trafficking in leishmania was shown to be essential for survival in macrophages [64]. Taken together, these findings suggest that leishmania may use clathrin-coated vesicles as a transport mechanism to direct vesicle trafficking at least, if not exocytosis of proteins from endosomal compartments to the extracellular milieu. Based on these findings, we propose that leishmania protein secretion probably involves the release of exosome-like vesicles, which may or may not be clathrin-coated. Moreover, we suggest that at least three distinct vesicular secretion processes contribute to the secretome, including exosomes, apoptotic vesicles, and glycosomes (Table 1).

Exosomes are small vesicles, 50 to 100 nm in diameter, which are released by fusion of either multivesicular endosomes or secretory lysosomes with the plasma membrane of eukaryotic cells [65-67]. Exosomes were initially described in reticulocytes as a mechanism for shedding organellar proteins and excess transferrin receptor during differentiation into mature nuclei free red blood cells [68]. Somewhat later, the proteomes of B lymphocyte and dendritic cell exosomes were described [24,38]. Dendritic cell exosomes have attracted a significant amount of attention because of their immunostimulatory properties as cell-free, peptide-based vaccines [69-72]. The striking correspondence between the leishmania secretome and these exosomes strongly suggests that protein secretion by leishmania involves the release of intraluminal vesicles originating from either the tubular lysosome [73] or multivesicular endosomes, or both. It is tempting to speculate that leishmania exosomes, like dendritic cell exosomes [69,70], may be capable of modulating the host immune response, although it likely that their properties may be quite distinct.

The formation of membrane blebs at the plasma membrane of apoptotic mammalian cells and their subsequent release are phenomena that have attracted significant attention [24,66,74]. As mentioned above, these apoptotic vesicles have been found to contain histone proteins and cytochrome c oxidase subunits. That leishmania undergo apoptosis is well established [25], and our finding that they release cytochrome c oxidase subunits and histones into Cm (Additional data files 1 and 5) suggests that they release apoptotic vesicles. Moreover, it has been shown that cultures of stationary phase leishmania promastigotes contain up to 43% apoptotic cells, and when the latter are removed by sorting the remaining nonapoptotic population is incapable of establishing and maintaining an infection [25]. These findings, taken together with our detection of apoptotic vesicle marker proteins, histones 1 through 4, in leishmania Cm (Additional data files 1 and 5), strongly suggest the possibility that leishmania apoptotic vesicles may be involved in pathogenesis. This could take the form of immune evasion, wherein (similar to activation of the 'silent phagocytosis' pathway used to internalize and clear very early apoptotic cells by mammalian macrophages [75]) these apoptotic vesicles would promote inhibition of macrophage activation before invasion by viable leishmania promastigotes.

Somewhat more difficult to explain from our findings is the suggestion for whole glycosome release, based upon both the characterized and the putative glycosomal proteins we detected in leishmania Cm (Table 1). Notably, many of the leishmania Cm proteins that were bioinformatically predicted to be glycosomal by the presence of PTS1 or PTS2 have been identified in purified glycosomes of the closely related kinetoplast *Trypanosoma brucei brucei *[76]. Our identification of the two most prevalent leishmania glycosomal membrane proteins in promastigote Cm (Additional data file 5) suggests that intact glycosomes were being exported from the cell. This is as opposed to a model in which these organelles were fusing with the flagellar pocket to release their cargo, in which case we would not have expected to have detected glycosomal membrane proteins *per se*. As we suggested above to potentially explain the secretion of translation machinery proteins, release of glycosomal proteins may be related to a stress response, but the targeted release of glycosomes with a more specialized function remains a possibility.

As previously stated, it is our hypothesis that the proteins with higher relative abundance in leishmania Cm are more likely to play an active role in pathogenesis than those proteins secreted to a lesser extent. Following this logic, export of proteins with lower Cm abundance may be related to either routine waste disposal or apoptotic blebbing, and these may be less likely to contribute to pathogenesis. Although this is a reasonable working model, it is not absolute and does not mean that proteins secreted in lesser abundance may not be of interest. In fact, EF-1α, a candidate virulence factor that has been shown to inhibit macrophage activation [17], had a Cm/CA peptide ratio in the lowest 20% of the ratio distribution (Figure 2, and Additional data files 2 and 5), and well below the cut-off for active secretion used to define the secretome. These data, especially when combined with the findings that apoptotic leishmania are required for leishmania disease development [25], support the interpretation that many of the proteins found in leishmania Cm are potential candidates for unique and essential roles in leishmania virulence, and further analysis will be required to prioritize those that should receive additional attention.

It should be mentioned that three leishmania proteins previously described to be secreted, namely SacP [14], chitinase [36], and silent information regulator (SIR)2 [18], were not identified in this LC-MS/MS analysis of leishmania Cm. One possible explanation for why these identifications were not made is that they have extremely low intracellular concentrations, with nearly all of the synthesized protein being secreted. Under these conditions other proteins present in the cell at a higher concentration could mask the CA peptide signals in the mass spectrometry. Importantly, the SILAC/mass spectrometry analysis was designed to compute ratios of simultaneously detected spectra from mixed Cm and CA samples. The absence of a CA signal in the mass spectrometry would have provided a denominator of zero, thereby not allowing for the computation of a meaningful Cm/CA ratio and exclusion from the analysis. Thus, no matter how abundant these peptides might be in Cm, without a comparable CA signal these proteins would not be included in the leishmania secretome, as defined by this study. It is possible that this explanation may also account for why chitinase and SIR2 were not identified in the secretome, especially considering that neither have characterized intracellular functions. Finally, we conducted these experiments using *L. donovani donovani*. Sequencing of the *L. donovani *genome is currently underway. As such we used the completed *L. major *genome to assign protein identities to the mass spectra gathered from leishmania Cm. Although the genomes for these two species are thought to be very similar, as their similar life cycles, biology, and expression profiles would indicate [77], it is possible that genomic difference between species prevented identification of some Cm proteins.

Examination of the secretome led to several additional findings worth noting. First, the number of proteins known to be associated with small vesicles outstripped by far the number of proteins identified that had classical secretion signals. This finding suggests that the main secretory route for leishmania involves the release of small vesicles. Second, for the majority of candidate virulence factors that were identified, it seems most likely that they may function to influence the survival of leishmania within the phagolysosome, although this remains to be formally tested. As the collection time for Cm was limited because of the need to culture organisms in the absence of serum, proteins in the secretome that may be involved in pathogenesis are likely to act during early stages of infection. During this early stage, they may contribute to the observed delay of phagosome maturation [78]. It has been proposed that delayed phagosome maturation represents a window of opportunity during which internalized promastigotes can differentiate into the more acid-tolerant amastigotes [79,80]. Whether the amastigote secretome is similar to or distinct from that of stationary phase promastigotes is not known at this time. However, given the relatively low stage-specific differences in gene expression that have been described [81], we do not regard significant differences to be likely. Third, targeting of virulence factors into host cell cytosol has been shown to be an effective strategy used by intracellular pathogens to remodel the environment and to influence host cell function [17,82-85]. After invading their macrophage hosts, leishmania have been shown to block cell activation, to inhibit microbicidal activity [86-88], and to attenuate antigen-presenting cell function [57,89,90]. A broad picture of the proteins secreted by leishmania in cell free culture provides a basis for investigation of effector proteins that may be active in host cells either within the phagolysosome or within host cytosol.

## Conclusion

This quantitative proteomic analysis identified a large and diverse pool of proteins in leishmania Cm and allowed us to define the leishmania secretome based on measurements of relative protein abundance in Cm that could only be explained by active secretion. The identities of proteins within the secretome revealed many candidates for further studies concerned with potential contributions to virulence and pathogenesis as well as to investigate mechanisms of secretion. Moreover, the data also indicate clearly that leishmania use predominantly nonclassical targeting mechanisms to direct protein export. This leads us to propose a model in which protein export occurs largely through the release of microvesicles, perhaps including exosome-like vesicles, apoptotic vesicles, and glycosomes.

## Materials and methods

### Cell culture

*L. donovani *Sudan strain 2S promastigotes were cultured in medium M199 supplemented with 10% fetal bovine serum (FBS; Gibco Cell Culture, Div. of Invitrogen Life Technologies, Gaithersburg, MD, USA), 1% penicillin and streptomycin, 20 mmol/l HEPES (Stem Cell Technologies, Vancouver, British Columbia, Canada), 6 μg/ml hemin, 2 mmol/l L-glutamine, 10 μg/ml folic acid, and 100 μmol/l adenosine at 26°C in a EchoTherm Chilling Incubator (Torrey Pines Scientific, San Marcos, CA, USA). Every third day the organisms were split 1:10 into fresh medium in 25 or 75 cm^3 ^cell culture flasks. For SILAC analysis, promastigotes were transferred to custom made M199 without L-arginine and L-lysine (Caisson Laboratories, North Logan, UT, USA) supplemented with 10% partially dialyzed FBS (Gibco), 1% penicillin and streptomycin, 20 mmol/l HEPES (Stem Cell Technologies), 6 μg/ml hemin, 2 mmol/l L-glutamine, 10 μg/ml folic acid, 100 μmol/l adenosine, and one of two SILAC media formulations (normal isotopic abundance arginine [42 mg/l] and lysine [73 mg/l]; and ^13^C_6_-arginine [43.5 mg/l] and ^2^H_4_-lysine [75 mg/l]) at 26°C. Organisms were cultured in this medium for at least 14 days and split 1:10 every third day, in order to achieve 100% labeling of cellular proteins before analysis. Stable isotope-labeled amino acids were purchased from Cambridge Isotope Laboratories (Andover, MA, USA). Except where otherwise noted, reagents were obtained from the Sigma-Aldrich Chemical Company (St. Louis, MO, USA).

### Isolation of promastigote Cm

Stationary phase promastigotes that had been grown either in medium containing normal isotopic abundance arginine and lysine or in medium containing ^13^C_6_-arginine ^2^H_4_-lysine L were collected by centrifugation at 300 × *g *for 10 minutes in a Beckman GS-6R centrifuge (Beckman-Coulter, Fullerton, CA, USA) and washed in Hanks balanced salt solution. Organisms were then concentrated tenfold by re-suspension in medium M199 without FBS and supplemented with 2 mmol/l L-glutamine, 10 mmol/l HEPES, 10 μg/ml soya bean trypsin inhibitor (Sigma-Aldrich), and either normal isotopic arginine and lysine or ^13^C_6_-Arg and ^2^H_4_-Lys in the concentrations given above for 4 to 6 hours at 26°C. Cm was isolated from cells by centrifugation at 300 × *g *for 10 minutes in a Beckman GS-6R. Supernatant was then subjected to centrifugation once more to ensure that no cells remained in suspension. Cm and cell pellets were either used immediately for enzymatic analysis or stored at -20°C for mass spectrometry analysis. A minimum of 5 × 10^8 ^promastigotes in culture was required to generate Cm with signals of adequate strength for mass spectrometry analysis.

Four times as many stationary phase organisms were required to generate sufficient Cm for detection of proteins by either metabolic labeling and autoradiography or by Western blotting. Two billion organisms were cultured in M199 containing normal isotopic arginine and lysine (Sigma-Aldrich). For autoradiography, cells were collected and washed as above, and then starved of methionine by resuspension in RPMI-1640 medium without methionine and cysteine (Sigma-Aldrich) with 1% FBS. After 1 hour 50 μCi/ml of ^35^S methionine (Sigma-Aldrich) was added and cells were cultured for a further 2 hours to allow labeling to occur. After washing to remove serum, cells were incubated for 4 hours in serum-free RPMI-1640 medium without methionine and cysteine, containing 10 mmol/l L-glutamine, 1 mmol/l HEPES, and 10 μg/μl Soya bean trypsin inhibitor, at which point the cells were separated from the Cm by low speed centrifugation to avoid mechanical lyses of cells. Pelleted cells were lysed on ice in lysis buffer (50 mmol/l Tris [pH 7.4], 1% Triton X-100, 0.15 mol/l NaCl, 1 mmol/l EGTA, 1 mmol/l phenylmethylsulfonyl fluoride, 10 μg aprotinin/ml, and 10 μg leupeptin/ml). Cell lysates were clarified by centrifugation in a microcentrifuge at maximum speed for 20 min at 4°C. The resulting WCL supernatants and the Cm were precipitated with trichloroacetic acid at 10% final concentration. The precipitates were solubilized in Laemmli sample buffer and equal counts/minute of Cm and WCL were separated by SDS-PAGE (5% to 20% gradient) followed by autoradiography.

For Western blotting, Cm was collected as above, but organisms were concentrated in normal isotopic M199. After separating Cm from the cells, WCLs were generated by sonicating the cell pellets to mimic lysis that may have occurred inadvertently during culture or centrifugation. Briefly, cell pellets were solubilized in 0.5 mmol/l Tris Laemmli sample buffer without SDS, bromophenol blue, or β-mercaptoethanol, but including protease inhibitors leupeptin and aprotinin both at 1 μg/ml and 10 μg/ml phenylmethylsulphonyl fluoride. The solution was sonicated three times at a power setting of 3 for 10 seconds. The lysate was cleared of insoluble material by centrifugation for 5 minutes at 10,000 × *g*. Following clarification the supernatant proteins were precipitated following the procedure bellow. The pellet was resuspended in Laemmli sample buffer without β-mercaptoethanol or bromophenol blue.

### Protein precipitation

For Western blotting and metabolic labeling analysis, proteins present within promastigote Cm were precipitated using pyrogallol red, as described previously [91]. Briefly, sodium deoxycholate was added to Cm to a final concentration of 0.02% and the solution was mixed for 30 minutes at 4°C to facilitate precipitation. Cm was then mixed with an equal volume of pyrogallol red solution (containing 0.05 mmol/l pyrogallol red, 0.16 mmol/l sodium molybdate, 1.0 mmol/l sodium oxalate, 50 mmol/l succinic acid, and 20% methanol [vol/vol]) and the pH adjusted to 2.0 with 2N HCl. The resulting solution was incubated at room temperature for 1 to 2 hours followed by 12 to 24 hours at 4°C. The Cm protein precipitates were harvested by centrifugation at 11,000 × *g *for 60 minutes at 4°C followed by two washes with ice cold acetone. The pellets were allowed to air dry before solubilization in Laemmli sample buffer without β-mercaptoethanol or bromophenol blue at 95°C for 30 minutes. Protein concentrations of the Cm and WCLs were measured using the BioRad DC Protein Assay (BioRad Laboratories Inc., Hercules, CA, USA).

### G6PD assay

Promastigote cell pellets were lysed by sonication to generate a WCL in 1 ml medium M199 with the appropriate concentrations of either normal isotope or nonradioactive isotope arginine and lysine, 1 mmol/l L-glutamine, 1 mmol/l HEPES, 10 μg/ml soya bean trypsin inhibitor, protease inhibitors leupeptin and aprotinin both at 1 μg/ml, and 10 μg/ml phenylmethylsulphonyl fluoride. After clearance by centrifugation at 11,000 × *g*, serial twofold dilutions of the lysate were made in medium M199 supplemented as above to yield final concentrations of 50%, 25%, 10%, 5%, and 1% (vol/vol). The concentrations of G6PD in 100 μl of Cm and in serial dilutions of WCL were assayed in 55 mmol/l Tris-HCl and 3.3 mmol/l MgCl_2 _buffer at pH 7.8, containing 3.3 mmol/l glucose-6-phosphate and 2 mmol/l NADP. Enzyme was obtained from the Sigma Chemical Company for a positive control. To generate a reference, 0.01 units of G6PD were stabilized in 5.0 mmol/l glycine with 0.01% bovine serum albumin (pH 8.0) and assayed along with sample and WCL dilutions. Enzyme reactions were carried out at 30°C and the change in absorbance, caused by changing NADP concentration, over 5 minutes was measured at 340 nm.

### LC-MS/MS of promastigote conditioned medium and data analysis

To identify proteins specifically secreted by leishmania into culture medium, direct quantitative comparisons of protein abundance in Cm versus CA were made on a protein-by-protein basis. The Cm was collected from leishmania grown in medium containing heavy isotopes of arginine and lysine, and compared with cell-associated material prepared from promastigotes grown in medium containing normal isotopic abundance amino acids. In some cases the reciprocal analysis was also carried out as well with identical results.

Approximately equal amounts of labeled and unlabeled protein (estimated from a preliminary LC-MS/MS analysis) from Cm and CA were mixed together and analyzed either by gel-enhanced LC-MS/MS exactly as described previously [92] or by peptide-level isoelectric focusing (IEF) combined with LC-MS/MS. For IEF, the protein mixture was solubilized in digestion buffer (50 mM NH_4_OH, 1% sodium deoxycholate, pH 8.0), denatured by heating to 99°C for 5 minutes, reduced by incubation with 1 μg dithiothreitol for 30 minutes at 37°C, alkylated with 5 μg iodoacetamide for 30 minutes at 37°C and finally digested by the addition of 1 μg porcine trypsin (Promega, Madison, WI, USA) overnight at 37°C. After digestion, the sample was acidified by addition of an equal volume of sample buffer (3% acetonitrile, 1% trifluoroacetic acid, and 0.5% acetic acid) and the deoxycholate that fell out of solution was pelleted at 16,100 × *g *for 5 minutes. Peptide mixtures were then desalted on STop-And-Go Extraction (STAGE) tips [93] before being resolved into 24 fractions from pH 3 to 10 on an OFFGEL IEF system (Agilent Technologies, Santa Clara, CA, USA), in accordance with the manufacturer's instructions. Fractions from the IEF were diluted with an equal volume of sample buffer, and each was desalted again on a STAGE tip. Each gel or OFFGEL fraction was analyzed on a linear trapping quadrupole-Fourier transform tandem mass spectrometer, as described previously [19]. Fragment spectra were extracted with ExtractMSN.exe (v3.2) using the default parameters (ThermoFisher Scientific, Ottawa, ON, CA); monoisotopic peak assignments were corrected with DTASuperCharge (default parameters [94]); and the resulting peak list was searched against the protein database for *L. major *plus the sequences of all human keratins and porcine trypsin (5 November 2006 version, 8,324 sequences) using Mascot (v2.1 [95]).

MSQuant [94] was used to parse Mascot result files, to recalibrate mass measurements, and to extract quantitative ratios. The final nonredundant list of proteins was generated using finaList.pl, an in-house script available on our website [96]. The false discovery rate for protein identifications based on two or more peptides with a measured mass accuracy under 3 ppm (the overall average was 0.61 ppm), a Mascot score of 25 or greater, and length 8 residues or more was estimated to be less than 0.5%, using reversed database searching. All identified peptides with their associated parameters can be found in Additional data file 1. SILAC ratios were extracted exactly as described previously [19]. The mean log_e _transformed ratios from four independent analyses and the relative standard deviations can be found in Additional data file 2.

### Western blotting

Following isolation of Cm, lysis of the corresponding cell pellet, and precipitation of proteins in both fractions, equivalent amounts of protein from the Cm and WCL were fractionated by SDS-PAGE. Proteins were transferred to nitrocellulose and probed with anti-EF-1α (Upstate Biotechnologies Inc., Lake Placid, NY, USA) following the manufacturer's instructions, as well as leishmania-specific antibodies to histidine secreted acid phosphatase [97] and against HSP70 and HSP90 [98] (a kind gift from Dr Joachim Clos).

### Scanning electron microscopy

Stationary phase promastigotes were washed in phosphate-buffered saline and fixed in 2.5% gluteraldehyde in 0.1 mol/l sodium cacodylate buffer (pH 7.2) containing 0.146 mol/l sucrose and 5 mmol/l CaCl_2 _at 22°C under vacuum in a microwave: 2 minutes at 100 W, 2 minutes without microwaves, 2 minutes with 100 W, and then repeated. Subsequently, fixed organisms were rinsed in the same buffer in the microwave for 40 seconds at 100 W two times and post-fixed in 1% OsO_4 _in 0.1 mol/l sodium cacodylate containing 2 mmol/l CaCl_2 _and 0.8% potassium ferricyanide (Polysciences, Warrington, PA, USA) at 22°C under vacuum in a microwave following the same steps used in the gluteraldehyde fixation. Cells were washed in distilled water at room temperature and allowed to adhere to poly-L-lysine (Sigma) coated coverslips. Subsequently the coverslips were dehydrated through an ascending ethanol series from 50% to 100%, each for 40 seconds at 100 W in a microwave. The fixed cells were critically point dried with liquid CO_2 _in a Balzars 020 Critical Point Dryer (Balzars Union Ltd, Lichtenstein) and coated with gold palladium using a Nanotech SEMPrep II sputter coater (Nanotech Ltd., Prestwick, U.K.). Samples were observed and imaged using a Hitachi S-2600 VPSEM (Hitachi High Technologies, Finchampstead, Wokingham, Berkshire, UK) at the University of British Columbia Bioimaging Facility.

### Bioinformatics screen of the genome of *Leishmania major *to identify candidate secreted proteins

The genome of *L. major *was accessed at the GeneDB *L. major *database [29]. Predictions of signal peptides and signal peptidase cleavage sites were made by SignalP [99]. Once these were provisionally identified, a filter was applied to remove those that contained more than one TM region predicted by TMpd [100]. Proteins with just one TM region were again screened to filter out those whose single TM domain did not overlap with the signal peptide coordinates. Finally, these putative classically secreted, non-TM proteins were screened for GPI attachment sites at the carboxyl-terminus using the GPI prediction program GPI-SOM [101].

### Gene Ontology

GO [30] annotations were performed using Blast2GO [31]. A nonredundant database was used as reference for Blastp searches with an expectation value minimum of 1 × e^-3 ^and a high scoring segment pair cut-off of 33. Annotations were made with default parameters. Briefly, the pre-eValue-Hit-Filter was 1 × e^-6^, the Annotation cut-off was 55, and the GO Weight was 5. The statistical framework GOSSIP [32] was used to identify statistically enriched GO terms associated with leishmania secreted proteins when compared to the GO terms associated with all of the proteins identified in leshmania Cm. GOSSIP generates 2 × 2 contingency tables for each GO term in the test group and uses a Fisher's exact test to calculate *P *values for each term. The *P *values are then adjusted for multiple testing by calculation of the false discovery rate and the family wise error rate [32].

### Statistical analysis

Statistical analyses of Cm/CA ratios and G6PD concentrations were performed using GraphPad Prism version 4.00 for Windows (GraphPad Software, San Diego, CA, USA).

## Abbreviations

CA, cell associated; Cm, conditioned medium; EF-1α, elongation factor-1α; FBS, fetal bovine serum; G6PD, glucose 6-phosphate dehydrogenase; GO, Gene Ontology; GPI, glycophosphotidylinositol; HSP, heat shock protein; IEF, isoelectric focusing; LC-MS/MS, liquid chromatography-tandem mass spectrometry; PTS, peroxisomal targeting signal; SacP, secreted acid phosphatase; SILAC, stable isotopic labeling of amino acids in culture; SIR, silent information regulator; TM, transmembrane; WCL, whole cell lysate.

## Authors' contributions

JMS was involved in study design, data collection and analysis, interpretation of results and manuscript preparation. SKC helped in design and implementation of the bioinformatics screen. DPR was involved in LC-MS/MS data collection and analysis. DD provided leishmania-specific antibodies to secreted acid phosphatase. DN helped with design of biochemical analyses. LJF was involved in study design, data collection and analysis, and manuscript preparation. NER was involved in study design, interpretation of results, and manuscript preparation.

## Additional data files

The following additional data files are available with the online version of this paper. Additional data file 1 is a table listing all the proteins, and the peptides contributing to their identification, detected in leishmania Cm. Additional data file 2 is a table showing a complete list of the SILAC ratios calculated for each Cm protein in each experiment, including the means of the four experiments. Additional data file 3 is a table listing all the GO terms associated with the leishmania Cm proteins. Additional data file 4 is a table listing the proteins predicted by bioinformatics to be secreted under the control of an amino-terminal secretion signal peptide; also shown here are the proteins with predicted GPI attachment sites and those proteins determined to be present in leishmania Cm by the SILAC LC-MS/MS analysis. Additional data file 5 is a table listing the leishmania Cm proteins, their mean SILAC ratios, and any documented microvesicle associations for these proteins.

## Supplementary Material

Additional data file 1 358 proteins had at least two nonoverlapping peptides that were detected and quantified in three or more individual analyses of leishmania Cm proteins. The peptides corresponding to each identification are shown. Protein identities were determined as described in Materials and methods and for Tables 1 to 3. Click here for file

Additional data file 2 After determining which proteins were to be considered for analysis (as described in Materials and methods and for Additional Data File 1), the measured Cm/CA ratios were normalized to the measured value of histone H2B in each independent experiment. The normalized values were then log normal (Ln) transformed (mean Ln transformed Cm/CA ratio, experiments [Exps] 1 to 4) to reduce the spread of the data. The means of the Ln transformed ratios for each protein identity were then calculated (mean Ln transformed values). The relative standard deviations of the peptide ratios for each analysis are included. Click here for file

Additional data file 3 GO annotation of the proteins detected in leishmania Cm. *Proteins with amino-terminal secretion signal peptides, and ^†^proteins shown to be antigenic. GO IDs lists the GO identification number associated with each protein, and GO Term lists the term associated with each GO ID. C, cellular compartment; F, molecular function; P, biologic process. Click here for file

Additional data file 4 Leishmania proteins predicted to be classically secreted by a genome wide screen for proteins containing an amino-terminal secretion signal peptide. MS, proteins detected in the SILAC/mass spectrometry analysis; § proteins detected by mass spectrometry with ratios above the secretome cut-off; GPI, proteins found to contain a GPI attachment site; *, proteins previously reported to be secreted by leishmania. Click here for file

Additional data file 5 Proteins with mean Cm/CA peptide ratios greater that two standard deviations above that of histone H2B were considered enriched. *Proteins with amino-terminal secretion signal peptides, and ^†^proteins shown to be antigenic. Microvesicle Association displays the vesicles associated with the protein ID. AP, adipocyte adiposome; BC, B-cell lymphocyte exosome; DC, dendritic cell exosome; Gly, glycosome. Click here for file
